# Temporary Ectopic Implantation of a Single Finger Using a Perforator as a Feeding Vessel, and Subsequent Prefabricated Chimeric Flap Transplantation

**Published:** 2012-02-23

**Authors:** Narushima Mitsunaga, Yamamoto Takumi, Hara Hisako, Yamamoto Yusuke, Oshima Azusa, Kikuchi Kazuki, Kato Harunosuke, Sata Kumiko, Doi Kentaro, Todokoro Takeshi, Araki Jun, Mihara Makoto, Higashino Takuya, Iida Takuya, Koshima Isao

**Affiliations:** Department of Plastic and Reconstructive Surgery, Tokyo University School of Medicine, Bunkyo-ku, Tokyo, Japan

## Abstract

**Objective:** Ectopic implantation was first reported by Godina in 1986. We herein present 2 cases in which amputated fingers were salvaged and reconstructed by means of temporary ectopic implantation utilizing perforator anastomoses and chimeric flaps. **Methods:** Case 1. A 30-year-old man injured his right hand. All of the fingers were completely crushed with the exception of the little finger. We performed an ectopic implantation by using the superficial circumflex iliac artery perforator. Three months later, the little finger was transplanted with the superficial circumflex iliac artery perforator flap, vascularized nerve, and the 2nd metacarpal bone. **Case 2.** A 29-year-old man suffered a degloving injury of the index finger. The digital artery was anastomosed to deep inferior epigastric artery perforator. One month later, a deep inferior epigastric artery perforator flap containing the ectopically transplanted index finger was transplanted, but the index fingertip became pale and necrotized. After debridement, a hemipulp transplantation was performed. **Results/Conclusions:** As the diameter of perforators is similar to that of digital arteries, and perforators are capable of supplying large areas of tissue, they can be used as recipient vessels for ectopic implantation in finger salvage procedures. Another advantage of perforators as feeding vessels in ectopic implantation is the possibility of forming an ectopic chimera; the finger can be incorporated as a part of the chimeric reconstructive flap. With respect to these advantages, the perforator can be used as a feeder in an ectopic implantation of single finger.

Replantation is the best choice for the survival of an amputated finger. However, the condition of the amputated finger stump often hampers replantation. Replantation of severely avulsed amputated fingers is associated with a high risk of failure. In these cases, temporary ectopic implantation can be used to salvage option of the amputated part.

Ectopic implantation was first reported by Godina, who implanted a subtotal amputated hand in the axilla in 1986.^1^ In 1996, Graf et al^2^ temporarily implanted ring and little fingers amputated at the level of metacarpophalangeal joints into the forearm. Salvage of amputated thumbs temporarily implanted into the ulnar and dorsalis pedis was reported by Li et al^3^ in 2008. We present 2 cases in which amputated fingers were salvaged and reconstructed by means of temporary ectopic implantation using perforator vessels and prefabricated chimeric flaps.

## CASE REPORTS

### Case 1

**Figure 1:**
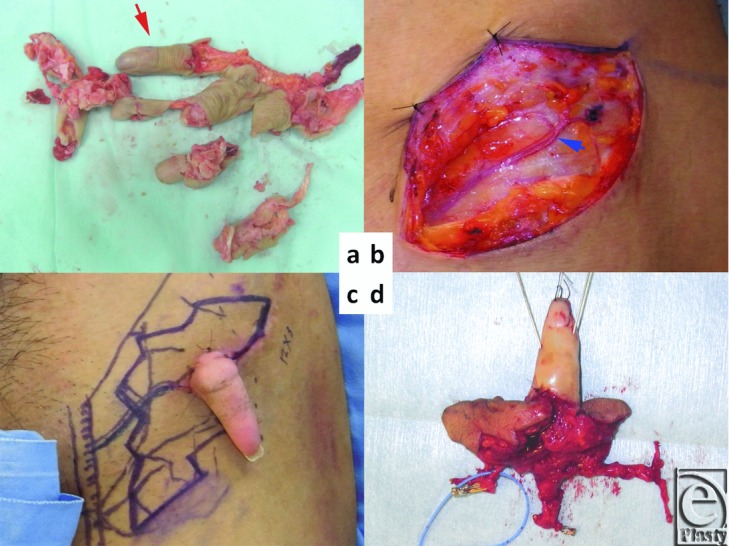
(a) Amputated little finger (arrow). (b) Left groin superficial circumflex iliac artery perforator (SCIP) (arrow). (c) Extopic chimeric flap design 3 months after ectopic implantation. (d) Chimeric SCIP flap with vascularized lateral femoral cutaneous nerve.

A 30-year-old man suffered a mangling injury of his right hand after it was caught in a meat grinder. All fingers were severely crushed with the exception of the little finger, which was intact distal to the proximal interphalangeal joint (Fig 1a). The hand was also crushed and ablated. We planned an ectopic implantation of the little finger to provide enough time to fully debride and optimize the hand in preparation for reimplantation of the little finger. The superficial circumflex iliac artery perforator (SCIP) and subdermal vein were first identified near the anterior superior iliac spine (Fig 1b). The diameter of the SCIP and the little finger digital artery were 0.4 mm and 0.6 mm, respectively. The digital artery of the little finger was anastomosed to the SCIP by using IVaS method.^4^

**Figure 2:**
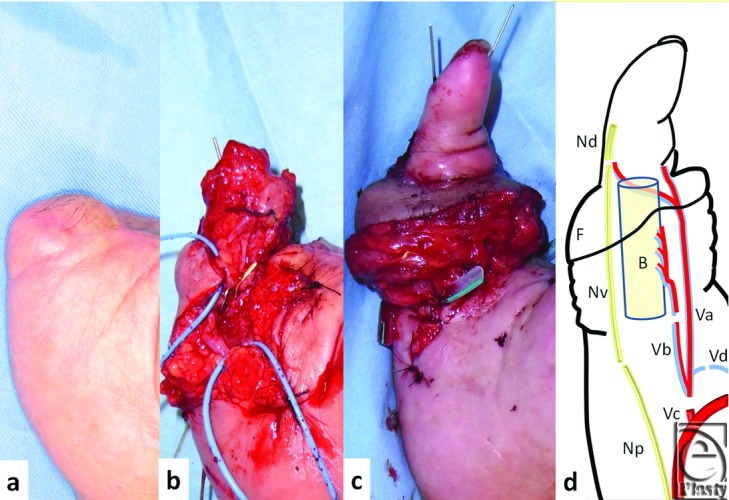
(a) Little finger 3 months after injury. (b) The right vascularized 2nd metacarpal bone was harvested on the 5th metacalpal bone for use as a proximal phalanx. (c) Amputated little finger with SCIP flap inset on the 2nd metacarpal bone. (d) Ectopic chimera transplantation. Nd indicates distal end of the 5th digital nerve; Np, proximal end of the 5th digital nerve; Nv, vascularized LFCN; Va, SCIA/SCIV; Vb, SIEA/SIEV; Vc, 5th common digital artery; Vd, subdermal vein.

Three months after ectopic implantation of the little finger, the little finger was elevated with an SCIP flap (12 × 3 cm), containing the lateral femoral cutaneous nerve (4 cm), a superficial circumflex iliac artery and vein (SCIA and SCIV), and superficial inferior epigastric artery (SIEA) (Figs 1c and 1d). Because the litter finger is shorter than a normal size finger, the right second metacarpal bone with a dorsal metacarpal artery was harvested and placed distally on the 5th metacarpal bone (Figs 2a and 2b). The SCIA and SIEA originate from a common trunk, which was anastomosed to the base of the 5th common digital artery. The dorsal metacarpal artery of the second metacarpal bone was anastomosed to the distal end of the SIEA (Fig 2d). The SCIV and subdermal vein of SCIP flap were anastomosed to the dorsal veins of the hand. The vascularized lateral femoral cutaneous nerve was anastomosed to both ends of the 5th digital nerve. Sensation in the little finger recovered, with a Semmes-Weinstein test result of 4.08 and moving 2 PD of 8 mm at 10 months, and SCIP flap also had sensation with a Semmes-Weinstein test of 4.31.

**Figure 3:**
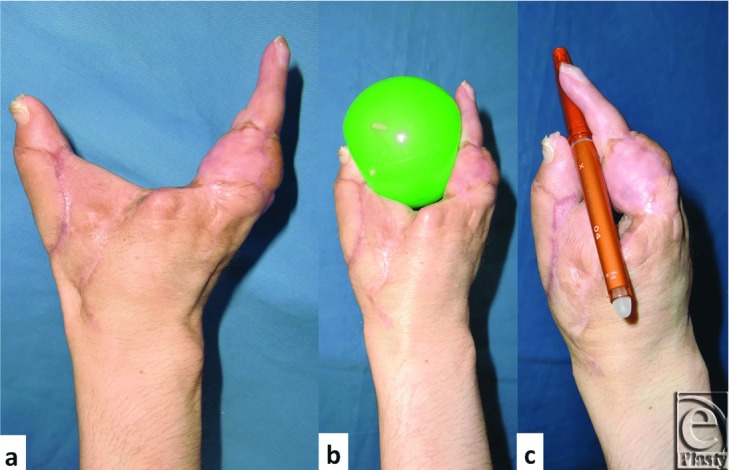
(a) The distance between the thumb and the little finger was 10 cm. (b) He is able to hold and throw a ball comfortably as well as (c) hold a pen.

Four months after transplantation of the little finger, toe to thumb transplantation was performed.^5^ (Figs 3a-3c). The maximum distance between the thumb and the little finger is 10 cm.

### Case 2

**Figure 4:**
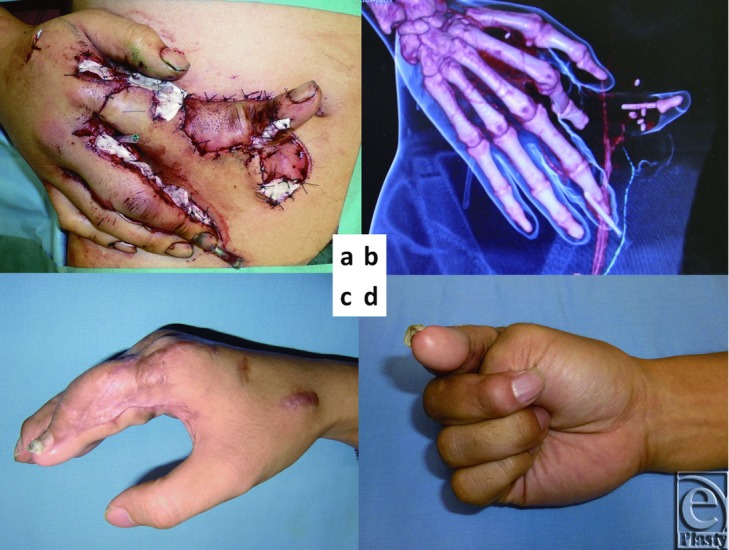
Amputation resulting from printing machine accident in a 29-year-old man. (a) The digital artery of the index finger was anastomosed to the deep inferior epigastric artery perforator (DIEP) at the level of the distal interphalangeal joint. (b) Computed tomographic angiography demonstrating connection between the DIEP and the digital artery. (c) Six months after ectopic implantation. (d) Grip position.

A 29-year-old man suffered an injury to his right hand after it was caught in a printing machine. The index finger was amputated at the distal interphalangeal (DIP) joint, and the skin was avulsed distal to the MP junction. The subdermal tissue of the amputated index finger was severely damaged. As immediate replantation seemed too hazardous, we opted for temporary ectopic implantation of the amputated part. The digital artery (diameter: 0.6 mm) was directly anastomosed to the right deep inferior epigastric artery perforator (DIEP/diameter: 0.8 mm) at the level of the DIP joint (Figs 4a and 4b). The digital nerve was marked with 6-0 nylon. The avulsed skin of the index finger was grafted onto the abdominal fat layer like a skin graft. The exposed bone and tendon of the proximal part of the index finger were covered with a pedicled DIEP flap. A month later, the chimeric DIEP flap with the index finger was harvested from the abdominal wall. The deep inferior epigastric artery and vein were anastomosed to the radial artery and the subdermal vein located in the snuff box.

Two days after this operation, the tip of the index finger became pale and partially necrotized. Index fingertip pulp was debrided and subsequently reconstructed with hemipulp transplantation from the great toe. The active postoperative motion was 0° of extension and 90° of flexion on the metacarpophalangeal joint of the injured index finger (Figs 4c and 4d). The proximal interphalangeal joint and DIP joint on the index finger were fixed. The uninjured fingers maintained their full range of motion.

The fingertip recovered good sensation. Semmes-Weinstein test was 2.83 and moving 2 PD was 8 mm after 2 years.

## DISCUSSION

Recently, applications for perforator flaps have expanded widely.^6^^,^^7^ Although the diameters of these perforators are very small, they are capable of perfusing large-volume flaps and tissues.

There are 3 advantages of perforators as recipient vessels in ectopic implantation. The first, the vessel size of the recipient is compatible with that of the donor site. Several arteries have been chosen as a feeding vessel for ectopic implantation, including the thoracodorsal, radial, ulnar, and dorsalis pedis artery.^1^^-^3 When compared with the distal part of the digital artery, however, these vessels have slightly larger diameters. Therefore, we utilized the DIEP and SCIP vessels, whose diameters of 0.8 mm and 0.4 mm were compatible with that of digital arteries (0.6 mm).

The second advantage of perforators is location. Deep inferior epigastric artery perforator and SCIP regions offer concealable scars with good cosmetic outcomes. Godina et al^1^ used the SCIA and SIEA as a recipient artery for his true first case of ectopic implantation in 1983.^1^ However, because the patient flexed his hip joint during sleep, it disrupted the anastomoses, and the fingers did not survive. If the finger had been placed more laterally as in an SCIP anastomosis, or more cranially as in a DIEP anastomosis, this complication would have been avoided.

Perhaps the greatest advantage of our method is the ability to prefabricate chimeric flaps following temporary ectopic implantation. We can elevate an ectopic finger with a perforator flap and a vascularized nerve with a sufficiently long pedicle as a chimeric flap at the second stage. Dissection of the initial digital artery anastomosis is not necessary during second operation as this would be extremely dangerous because of surrounding fibrosis. Nazerani and Motamedi^8^ reported a “piggyback” method for ectopic single-fingertip transplantation. In this method, the transplanted part is attached to the amputation stump like a pedicle flap after ectopic implantation of fingertip to groin. After a 3- to 4-week delay, the finger/groin flap composite flap is divided and transferred to amputation stump without anastomosis of the vessels. The indication of this method is for fingertip amputation of zone 1 and zone 2a. Our case 2 might be able to use this technique. However, this method is unsuitable for long fingers, and no additional tissues can be harvested for reconstruction. In our method, Ectopic Chimeric flap transplantation can be performed with decreased risk of damage to the feeding vessels and reconstruction of missing components in cases of partial finger defects.

Disadvantages to this technique include limited suitable perforators. However, because multiple perforators (DIEP, SCIP, SIEA, and ICAP) exist in the groin and lower abdominal area, alternative perforators can be localized and utilized if one perforator is deemed unsuitable.

## CONCLUSION

We herein described 2 cases using perforators as recipient vessels for temporary ectopic implantation. The diameters of these perforators were suitable for anastomosis with the digital artery.

This method may be able to salvage severely crushed fingers with partial soft tissue or bony defects, which would otherwise have been deemed not replantable.
